# The effect of dietary supplementation on brain-derived neurotrophic factor and cognitive functioning in Alzheimer's dementia

**Published:** 2017-12-03

**Authors:** Alicia Martin, Jordan Stillman, Maria-Jose Miguez, H. Reginald McDaniel, Janet Konefal, Judi M. Woolger, John E. Lewis

**Affiliations:** ^1^Departments of Psychiatry & Behavioral Sciences, University of Miami Miller School of Medicine, Miami, FL, United States; ^2^School of Integrated Sciences and Humanity at Florida International University, Miami, FL, United States; ^3^Fisher Institute for Medical Research, Grand Prairie, TX, United States; ^4^Department of Family Medicine & Community Health, University of Miami Miller School of Medicine, Miami, FL, United States; ^5^Medicine, University of Miami Miller School of Medicine, Miami, FL, United States

**Keywords:** Alzheimer's dementia, brain-derived neurotrophic factor, aloe polymannose, cognitive function, dietary supplementation

## Abstract

**Background and Aim:**

The objective of the present study was to investigate the effect of an aloe polymannose multinutrient complex (APMC) on pro brain-derived neurotrophic factor (BDNF) and mature BDNF in persons with moderate to severe Alzheimer's dementia.

**Methods:**

A 12-month open-label trial was utilized to evaluate the effect of the APMC on proBDNF and BDNF and their relationship to cognitive functioning. Thirty-four adults were enrolled and consumed 4 teaspoons/day of APMC for 12 months. Subjects were assessed at baseline and twelve months follow-up for proBDNF and BDNF and with a neuropsychological battery to measure cognitive functioning. Cognitive functioning was correlated with proBDNF and BDNF.

**Results:**

Few adverse effects were reported. While proBDNF (baseline M = 6,108.9, SD = 854.9 and 12 months M = 5,799.2, SD = 573.4; *p* = 0.57) and BDNF (baseline M = 5,673.8, SD = 3,342.3 and 12 months M = 6,312.9, SD = 2,830.9; *p* = 0.29) did not significantly change, the correlations between the ADAS-cog total score and BDNF (r = -0.53, *p* = 0.04) and BDNF/proBDNF ratio (r = -0.58, *p* = 0.05) became statistically significant after 12 months of dietary supplementation. Other correlations were noted for various cognitive functioning assessments and BDNF and/or BDNF/proBDNF ratio at both baseline and 12 months.

**Conclusions:**

These findings suggest that the relationship between cognitive functioning and BDNF and BDNF/proBDNF ratio improved in response to consumption of a dietary supplement in persons with Alzheimer's dementia, which is consistent with our previous findings on cognitive functioning.

**Relevance for patients:**

Overall, our results showed modest improvements in clinical outcomes for a disease that otherwise has no standard conventional approach to treatment with proven efficacy.

## 1. Introduction

In 2015, Alzheimer's dementia (AD) cost roughly $226 billion in direct treatment and without improvements in existing modalities it is projected to cost over $1.1 trillion in 2050 [1]. Treatment options for AD are extremely limited and are mostly given to address co-morbid symptoms, such as sleeplessness, agitation, wandering, anxiety, and depression [2,3]. AD is characterized by a progressive synaptic and dendritic loss with associated neuronal dysfunction and death, leading to a clinical decline [4,5].

Since neurotrophic factors, particularly brain-derived neurotrophic factor (BDNF), play a critical role in neurosynaptic function, apoptosis, plasticity, long-term potentiation, learning memory processes, and higher order thinking [6-11], BDNF has been recognized as a desirable target for treatment of AD. BDNF is produced as pro-isoforms that are cleaved to release the mature factor [9,12]. For decades, the assumption was that proBDNF did not have a physiological function, but very recently Yang and colleagues demonstrated that proBDNF interacts with the low-affinity receptor p75, while mature BDNF signals through its high-affinity tropomyosin-related kinase B (TrkB) receptor [13]. As a result, they frequently exert opposite neuronal functions [14]. Therefore, it is critical to measure both and the ratio between the two when studying their potential role on cognitive function and dementia.

Utilizing dietary supplements may offer a way to ameliorate some of the symptoms associated with AD, while also simultaneously enhancing BDNF levels. Our group conducted an open-label, pilot study of 48 AD patients that showed improvements in quality of life according to an Alzheimer's Association modified symptoms checklist after the subjects consumed an aloe-based dietary supplement for 6 months [15]. Next, we conducted a 12-month intervention in persons with moderate to severe AD showing that an aloe polymannose multinutrient complex (APMC) improved cognitive function (statistically and clinically significant increase according to the ADAS-cog cognition score) [16]. While the clinical improvement was supported by the enhancements in the serological data, no neurological mechanism of action was established in the study. Additionally, animal studies and at least one human study have shown that many of the nutrients in APMC, including polysaccharides, antioxidants, resveratrol, and omega-3 fatty acids, have individual benefits on cognition and memory [17-21] and can increase BDNF levels [22-25]. The sum of these findings suggests that long-term oral supplementation can modulate BDNF production in the brain and can also explain the memory improvements demonstrated in our prior studies.

In accordance with this view, we postulate that part of the benefits of APMC could be the result of improvements in BDNF. Thus, in the current study, our primary objectives are: (1) since inducing endogenous rather than exogenous production may be a useful way to improve cognitive function, we will assess the effect of APMC on proBDNF and BDNF from baseline to after 12 months of supplementation; (2) determine if proBDNF and BDNF are correlated with any of our assessments of cognitive functioning; and (3) considering that no desirable peripheral level of BDNF has been established, we would like to confirm if the 5,000 pg/mL cutoff value that we found relevant to discriminate between subjects with and without cognitive impairments among people living with HIV will be applicable/useful in the current target population. These objectives will allow us to evaluate BDNF as a possible mechanism of action to at least partially explain improvements in cognitive functioning due to supplementation with APMC.

## 2. Methods

*Study participants.* Participants (n = 34) were recruited from the Miami Jewish Health Systems outpatient facility from 2008 to 2011. The study was conducted with the approval of the Stein Gerontological Institute Institutional Review Board for human subjects research, and each subject and the primary caregiver signed informed consent before participating in the study. This study was not listed with clinicaltrials.gov, as that was not a policy requirement at that time. Inclusion criteria were: (a) >60 years of age, (b) a clinical diagnosis by the study psychiatrist of probable moderate-to-severe AD for at least one year, (c) a score of between 10 and 26 on the Mini-Mental State Examination (MMSE) [26], (d) sufficient vision and hearing to comply with study procedures, and (e) able to provide informed consent by the subject or primary caregiver. Exclusion criteria were: (a) diagnoses of delirium or depression, (b) currently participating in another nutritional supplement study, and (c) a known allergy to eating shrimp. Each participant was evaluated by the study psychiatrist prior to enrollment in the study to verify the diagnosis of AD according to the standards and criteria of the Diagnostic and Statistical Manual of Mental Disorders. Subjects were not required to change their medication regimen to participate in the study and continued to take their daily medication as prescribed by their treating physician.

*Intervention.* The polysaccharide-based multinutrient formula used in this study is a nutritional supplement that has been sold by several commercial entities for over 20 years. According to the company's literature (www.wellnessquest.org), APMC contains stabilized rice bran, larch tree fiber and larch tree soluble extract, cysteine, soy lecithin, aloe leaf powder (BiAloe™), inositol hexaphosphate, dioscorea powder, omega-3 spherules, citric acid, glucosamine, cherry tart powder, and ultraterra calcium alumino silicate. The final product is a powder, packaged in 300-gram containers, which dissolves readily in any liquid. All participants consumed 1 teaspoon orally of the APMC four times per day (with 3 meals and before bedtime). The primary caregiver was shown how to administer the APMC at the baseline assessment, and the first dose was given to the participant at our facility to ensure compliance with the method and to monitor for any complications or adverse effects.

*Assessment schedule.* Each participant's caregiver completed a basic demographics and medical history questionnaire at baseline. In addition, a neuropsychological battery of three measures was administered by a neuropsychologist who was very experienced in working with the elderly and dementia patients to assess fluctuations in cognitive functioning, disease severity, daily life activities, and quality of life. The neuropsychological battery required no more than 90 minutes to complete. A standard assessment at 12-month follow-up was conducted to monitor: (a) adverse reactions and compliance to the intervention, (b) current medications, and (c) basic medical and health status. At baseline and 12 months, a blood sample was drawn to assess changes in BDNF and proBDNF.

*Cognitive functioning.* The Alzheimer's Disease Assessment Scale-Cognitive Score (ADAS-cog) [27] is a sensitive and reliable psychometric scale, which is considered to be the benchmark measure to assess cognitive functioning in dementia studies [28]. It has eleven subscales that evaluate orientation, attention, memory, language, reasoning, and constructional and ideational praxis that are summed up to create a total cognition score [29]. The total score can range from zero (no impairment) to 70 (severe impairment). Different, counterbalanced word lists were used at the follow-up visits to ensure that practice and carry-over effects would not confound our results. The ADAS- cog assessment included an additional concentration score with values ranging from zero (no impairment) to 5 (severe impairment). In addition to the ADAS-cog, the Severe Impairment Battery (SIB) [30,31] is a 40-item questionnaire designed to assess the severity of cognitive dysfunction in AD and is divided into nine domains: memory, language, orientation, attention, praxis, visuospatial, construction, orientation to name, and social interaction. The total score on the SIB ranges from zero (greatest impairment) to 100 (no impairment). The MMSE is one of the most widely utilized and popular brief cognitive assessments, providing a rapid screen of orientation, registration, attention and calculation, recall, and language domains [26]. The score can range from zero to 30 (25+ is normal) and can indicate severe (<9 points), moderate (10-20 points), or mild (21-24 points) cognitive impairment [32].

*BDNF and proBDNF sample collection and processing. *Venous blood was obtained at two different time points (baseline and 12 months) from all participants on the same day that they came to the center for cognitive assessments. Circulating levels of BDNF were selected because prior studies have demonstrated that, although different from those in the cerebrospinal fluid (CSF), they are correlated with CSF measures in other central nervous system (CNS) diseases [33]. To obtain platelet-poor plasma (PPP), blood samples were collected in EDTA-coated tubes (plasma) (BD Diagnostics, Franklin Lakes, NJ), stored on ice, and delivered to the laboratory within two hours of data collection. Plasma was separated by centrifugation at 40° C for 15 minutes at 1,500x g. This plasma was again re-centrifuged at 10,000x g, and aliquots of PPP were stored in polypropylene tubes at -80° C until assayed. PPP BDNF and proBDNF levels were measured using a commercially available ELISA kit (R&D System) according to the manufacturer's instructions and were calculated based on a standard curve. The minimum detectable concentration of BDNF is typically less than 62 pg/mL. The repeatability of the BDNF ELISA, as measured by intra-assay precision, was 6%, and the reproducibility as measured by inter-assay precision was 9%. Coefficient of variation was 7.9 (CV% = SD/mean* 100%).

*Statistical analysis.* Data were analyzed using SPSS 24 (IBM Inc., Chicago, IL) for Windows. Frequency and descriptive statistics were calculated on all variables. We utilized paired samples t-tests to assess changes in BDNF, proBDNF, and BDNF/proBDNF ratio from baseline to 12-month follow-up. We examined the relationships between BDNF, proBDNF, and BDNF/proBDNF ratio and the cognitive assessments (total scores and individual domains on each measure) at baseline and 12 months follow-up with Pearson product-moment correlations. We calculated differences in baseline and 12-month values on BDNF, proBDNF, and the cognitive assessments to assess for correlation among the difference scores. We split the sample by BDNF at 5,000 pg/mL based on our prior findings to assess differences in cognitive functioning between the two groups. The criterion for statistical significance was a = 0.05.

## 3. Results

*Compliance to the intervention.* Caregivers of the participants were asked at each follow-up visit to return containers to check for the amount left over and to confirm compliance to the protocol. Also, study staff periodically called the caregivers between assessments to ensure that the protocol was being followed and to enhance rapport between the study team and the participants. Caregivers were unanimously noted to have followed the protocol, as they were all highly motivated to participate in a study that might offer a possibility of some level of improvement in the participant's quality of life.

*Sociodemographics.* The sample comprised of 82% females (n=28) and 18% males (n=6) with a mean age of 79.9±8.4 years. The racial/ethnic distribution of the subjects was as follows: 62% Hispanic (n=21), 29% white, non-Hispanic (n=10), and 9% black, non-Hispanic (n=3). See Table 1 for all sociodemographic characteristics of the sample. At baseline, BDNF and proBDNF were not correlated with age, sex, race/ethnicity, marital status, educational attainment, or years diagnosed with AD. 

**Table 1.**
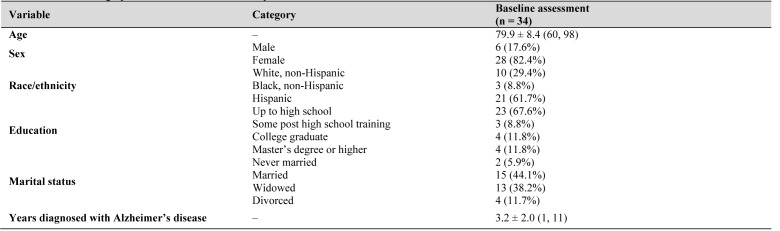
Sociodemographic characteristics of the sample.

**Figure 1.**
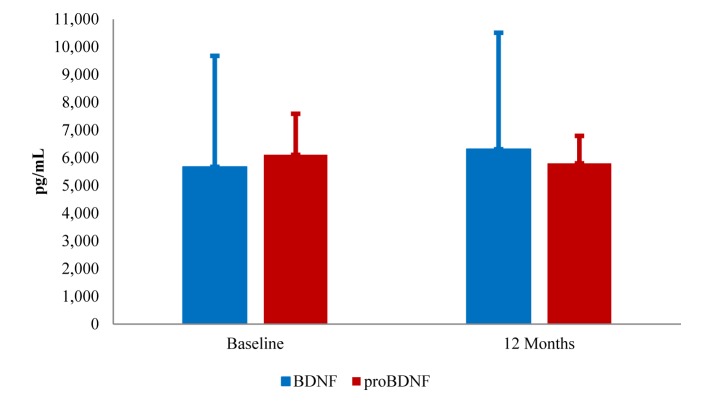
BDNF and proBDNF at baseline and 12 months follow-up. *NOTE: Data are mean ± standard deviation.*

*The effect of APMC on BDNF and proBDNF.* Descriptive statistics for BDNF and proBDNF before and at the end of experimental treatment are displayed in Figure 1. Although BDNF increased at 12 months, the change was not statistically significant (*p* = 0.29), and proBDNF decreased slightly at the end of treatment (*p* = 0.57). The BDNF/proBDNF ratio (p=0.48) remained relatively constant for the duration of the intervention (baseline: 1.0 ± 0.7 vs. 12 months: 1.2 ± 0.6).

*Cognitive functioning.* The baseline ADAS-cog Total Cognitive Score did not differ by sex (males: 36.7 ± 15.9 vs. females: 43.1±13.6, p=0.32) or by educational attainment (<2 years: 41.7±12.5 vs. >12 years: 42.7 ± 17.4, *p* = 0.84). The baseline SIB Total Score was also similar for sex (males: 66.7 ± 37.6 vs. females: 56.2 ± 30.4, *p* = 0.47) and educational attainment (<12 years: 58.1 ± 29.6 vs. >12 years: 58.0 ± 36.5, *p* = 0.99).

*The relation between BDNF and cognitive functioning.* At baseline, BDNF was correlated with the ADAS-cog Spoken Language Ability score (r = -0.41, *p* = 0.05), ADAS-cog Comprehension score (r = -0.53, *p* = 0.01), the ADAS-cog Concentration/Distractibility score (r = -0.48, *p* = 0.02), the SIB Social Interaction total score (r = 0.43, *p* = 0.04), the SIB Construction total score (r = 0.40, *p* = 0.05), and the SIB Orienting to Name score (r = 0.42, *p* = 0.04). At follow-up, an inverse correlation between BDNF and the ADAS-cog Total Cognitive score at baseline was evident after 12 months of dietary supplementation (r = -0.53, *p* = 0.04). BDNF was also correlated with the ADAS-cog Word Recognition score (r = -0.85, p < 0.01). No significant correlations were noted for proBDNF.

*The relation between BDNF/proBDNF ratio and cognitive functioning.* The BDNF/proBDNF ratio was not correlated with the ADAS-cog Total Cognitive score at baseline (r = -0.46, *p* = 0.14), but became significant at 12 months follow-up (r = -0.58, *p* = 0.05). Additionally, at baseline, the BDNF/proBDNF ratio was negatively correlated with the Comprehension score (r = -0.81, *p* < 0.01) and the Concentration /Distractibility score (r = -0.59, p < 0.05) on the ADAS-cog. At follow-up, the BDNF/proBDNF ratio was correlated with the ADAS-cog Word Recognition total reminders (r = -0.82, *p* = 0.04). No other correlations were significant.

*The relation between changes in BDNF, proBDNF, and cognitive functioning.* We calculated changes in BDNF, proBDNF, the ADAS-cog, and SIB to evaluate correlation in the difference scores. We found a significant relationship between changes in ADAS-cog Word-Finding Difficulty in Spontaneous Speech and BDNF (r = -0.45, *p* = 0.02).

**Table 2.**
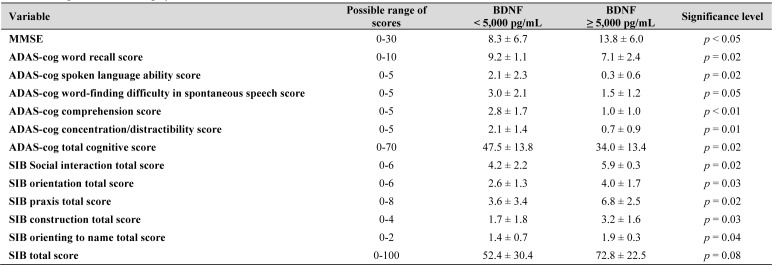
Cognitive functioning by BDNF level.

## 4. Discussion

As previously mentioned, proBDNF and BDNF play critical roles in neurosynaptic function, apoptosis, plasticity, long-term potentiation, learning memory processes, and higher order thinking [6-11]. More specifically, BDNF is active in the synapses of the CNS, where it works to promote growth, maturation, neuronal survival, and synaptic plasticity, which affect learning and memory.

With very limited options for prevention or treatment of AD, any intervention that demonstrates promise for improving the patient's condition is needed to reduce the burden on victims of AD and their caregivers. Additionally, the ever-increasing financial burden of AD and other chronic diseases puts stress on the aging United States population and the healthcare system that supports it.

Given that experimental pharmacological trials continue failing to offer ameliorative remedies for this disease, alternatives such as nutrition and dietary supplement approaches may prove to be beneficial. We previously showed that this sample of participants had clinically and statistically significant improvements in cognitive functioning at 12 months on the ADAS-cog total cognition score in response to APMC [16]. Even with the significant clinical finding, no neurological mechanism of action was identified to support it.

The Stratification of BDNF to Assess Cognitive Functioning. Based on our prior work, we dichotomized participants based on BDNF levels above and below 5000 pg. Those subjects below 5000 pg scored worse than those subjects at or above 5000 pg on several cognitive tests, including the MMSE (p < 0.05) and the ADAS-cog Total Cognitive score (p = 0.02). See Table 2 for the complete list of significant findings

As a logical next step, we focused on elucidating the potential mechanisms of action and proposed BDNF/pro-BDNF as a neural mechanism of action. Our findings at 12 months not only support previous research that BDNF plays a fundamental role in memory, behavior, and cognition [1,34-44], but also suggests that our dietary supplementation may have had a positive effect on BDNF levels, which affected cognitive functioning. These results are also consistent with our previous findings that cognitive functioning improved at 12 months, where we first theorized that the improvement could have been due to both a proliferation of CD14+ cells and reductions in neuroinflammation [16]. The current findings suggest a potential mechanism involving BDNF that may contribute to improved cognitive functioning. Additionally, we showed at baseline that those at BDNF <5000 pg/mL performed worse on several of the cognitive assessments than those that were BDNF >5000 pg/mL. We stratified participants at 5000 pg/mL because our prior findings suggested that this threshold distinguishes cognitive functioning to some degree in persons with HIV. As noted in the current study, cognitive performance was better in the group at 5000 pg/mL or higher for the MMSE, every ADAS-cog scale, and all SIB scores, except for the SIB total score. The MMSE provides a quick evaluation of cognitive function, including orientation, registration, attention and calculation, recall, and language domains. Compared to the MMSE, the ADAS-cog is a more comprehensive assessment of cognitive function that also include portions of the test devoted to orientation, attention, memory, language, reasoning, and constructional and ideational praxis. Similar to the ADAS-cog, the SIB comprehensively assesses cognitive function through the same domains plus social interaction. Thus, whether a quick, simple assessment (MMSE) or a more comprehensive evaluation (ADAS-cog and SIB), a higher BDNF level distinguished better cognitive function in our sample. These results further support the notion that the level of BDNF may be an important factor in determining clinical outcomes for AD patients.

## 5. Limitations

We note several limitations of the current investigation. We did not assess dietary intake, physical activity, depression, anxiety, or caregiver support, so we do not know how these factors may have altered our findings. The neuropsychologist was unblinded to the study participants, but she assessed subjects for all studies occurring simultaneously at our center, so her influence, if any, on this study should have been systematic. We were limited by a small sample, so more participants could potentially yield even more fruitful results. We did not limit the study by medication use, given the obvious ethical considerations associated with this disease.

## 6. Conclusions

The development of an effective approach for the treatment of AD is thus of major importance, as current strategies are limited to agents that attempt to attenuate disease symptomatology without addressing the causes of disease. Our results compel further study with a larger sample size and more frequent assessments of BDNF and proBDNF to better evaluate their relationship to cognitive functioning in response to the APMC formula over an extended period of time. Using this type of model would allow us to gain deeper, if not novel, insights into the pathophysiology of a disease that is the source of much human suffering.
